# Proprioception and muscle performance unchanged by in-home step training in multiple sclerosis: secondary outcomes analysis

**DOI:** 10.7717/peerj.20354

**Published:** 2025-12-19

**Authors:** Zoë J. Djajadikarta, Siobhan C. Dongés, Joanna Diong, Phu D. Hoang, David S. Kennedy, Jasmine C. Menant, Stephen R. Lord, Janet L. Taylor, Simon C. Gandevia

**Affiliations:** 1 Neuroscience Research Australia, Sydney, NSW, Australia; 2 School of Medical Sciences, Faculty of Medicine and Health, The University of Sydney, Sydney, NSW, Australia; 3 University of New South Wales, Sydney, NSW, Australia; 4 Graduate School of Health, Physiotherapy, University of Technology Sydney, Sydney, NSW, Australia; 5 Motion and Mobility Research Laboratory, University of Victoria, Victoria, British Columbia, Canada; 6 Discipline of Exercise and Sports Science, Edith Cowan University, Perth, WA, Australia

**Keywords:** Multiple Sclerosis, Proprioception, Neurophysiology, Muscle Contraction, Muscle Fatigue, Ankle, Exergame, Training

## Abstract

**Background:**

The Interactive Step Training to Reduce Falls in People with Multiple Sclerosis (iFIMS) trial was a multi-centre, parallel-designed, randomised controlled trial testing an in-home, computerised exergame playing system (*smart±step*) in people with multiple sclerosis (Expanded Disability Status Scale 2–6).

**Objective:**

This study was nested within the iFIMS trial to assess whether the *smart±step* system could improve secondary outcomes of ankle proprioception and plantarflexor muscle performance.

**Methods:**

Tests of ankle proprioception and plantarflexor muscle performance were performed before and after 6 months of intervention with the *smart±step* system (intervention group; *n* = 33), or 6 months of usual care (control group; *n* = 33). Ankle proprioception outcomes included movement detection threshold and reaction time. Plantarflexor muscle performance outcomes included maximal voluntary torque, twitch torque from electrical stimulation, voluntary activation (level of neural drive), decrease in these parameters after a 2-min sustained isometric contraction, and time-to-recovery of these parameters.

**Results:**

There were no differences between the intervention and control groups for all proprioception and muscle performance outcomes (95% CI of mean differences crossed 0), and no difference in time-to-recovery after the sustained contraction (95% CI of hazard ratios crossed 1).

**Conclusions:**

The *smart±step* system did not improve proprioception or muscle performance over a 6-month intervention, compared to usual care, in people with multiple sclerosis. However, at-home interventions are cost effective and convenient, and the *smart±step* system could help maintain physical activity in an engaging way in this group.

## Introduction

People with multiple sclerosis (MS) fall on average more than once per month (Nilsagård et al., 2015). Falling is associated with injuries, subsequent fear of falling, mobility impairment and reduction in activity (*e.g.*, Matsuda et al., 2011; Matsuda et al., 2012; Gunn et al., 2014; Peterson, Cho & Finlayson, 2007). Measures of impaired stable standing and walking, including reduced walking speed, poor standing and leaning balance and slow stepping reaction times, are among the predictors of falls (*e.g.*, Hoang et al., 2014; Giannì et al., 2014; Coote et al., 2020). In a pilot randomised controlled trial, 12 weeks of step training in people with MS reduced factors that contribute to the risk of falling (Hoang et al., 2016). The changes included improvements in standing balance, stepping, coordination and functional performance. Standing, stepping and walking are coordinated sensorimotor tasks and it is not clear whether the benefits of step training occur primarily through improvements in sensory function, motor function and/or in the reflex and cognitively controlled interactions between them.

The senses of ankle position and movement (aspects of ankle proprioception) (Proske & Gandevia, 2012) and plantarflexor muscle performance are two important contributors to standing balance and walking (*e.g.*, Di Giulio et al., 2009; Neptune, Kautz & Zajac, 2001; Tavakkoli Oskouei et al., 2021). In MS, proprioceptive deficits and slowed central conduction are common, and are associated with impaired balance and walking (Cameron et al., 2008; Sato et al., 2021; Whittier & Bandera, 2020). Decreased strength and endurance of the ankle plantarflexors are also common and can also substantially impair balance and gait (Mañago et al., 2018; Massot et al., 2021). Damage to the central nervous system impairs central neural drive (voluntary activation) to the muscles, (Capone et al., 2020; Mamoei et al., 2020) which can result in this reduced muscle strength and endurance.

In the general population, exercise can improve performance of sensorimotor tasks including balance, as well muscle strength and endurance through muscle and neural adaptations (*e.g.*, Bakker et al., 2024; Behm et al., 2015). In people with multiple sclerosis, exercise training also improves walking and balance, (Gunn et al., 2015; Snook & Motl, 2009) although it is less clear that it improves falls (Gunn et al., 2015). Meta-analyses suggest that, for people with MS, general exercise training and specific gait, balance and functional training lead to improvements in standing balance (15 studies; Gunn et al., 2015) and that resistance or aerobic exercise training can lead to small improvements in walking and mobility (22 studies; Snook & Motl, 2009).

We aimed to investigate whether step training in people with MS improved ankle proprioception and muscle performance. This study was nested within a larger clinical trial called: An Interactive Step Training System to Reduce Falls in People with Multiple Sclerosis (iFIMS) (Hoang et al., 2024). This was a multi-centre randomised controlled trial of the *smart*±*step* in-home computerised game playing system; an interactive, step-training system that requires a combination of stepping and balancing to play computerised games, known as exergames (Sturnieks et al., 2019). Following a pilot trial that showed that 12 weeks of step training improved factors that contribute to the risk of falls (Hoang et al., 2016), iFIMS was designed to determine whether step training decreased falls. It investigated the effect of a 6-month step-training program using *smart*±*step* on the frequency of falls in people with multiple sclerosis compared to 6 months of usual care. Secondary outcomes comprised stepping, gait, balance, strength, and cognitive performance measures (Hoang et al., 2024).

In the present study, we investigated the effects of 6 months of *smart*±*step* step training on ankle proprioception and muscle performance in a subgroup of people undergoing the main trial. We hypothesised that the intervention would improve ankle proprioception and/or plantarflexor muscle performance in people with multiple sclerosis.

## Materials & Methods

### Trial design

This study was an assessor-blinded, parallel, randomised controlled trial. Participants were allocated to the intervention or control arm in a 1:1 ratio. The study was registered with the main iFIMS trial prior to recruitment of the first participant (ANZCTR trial registration number ACTRN12616001053415). Protocols and statistical analysis plans were publicly registered with Open Science Framework prior to data analysis (Djajadikarta, 2020; Menant et al., 2020) and the study was reported in line with CONSORT guidelines (CONSORT checklist: Supplemental 1) (Schulz et al., 2010).

### Participants

This trial was approved by the Human Research Ethics Committees of South Eastern Sydney Local Health District (HREC 14/312) and the University of New South Wales (HREC number: HC14211) and was conducted according to the Declaration of Helsinki. All participants provided written, informed consent prior to their involvement.

Participants were included if they: were community-dwelling adults (aged ≥ 18 years), had been diagnosed with MS, had an Expanded Disability Status Scale (EDSS) (Kurtzke, 1983) score between 2.0–6.0, had no apparent cognitive impairment (*i.e.,* were able to understand and follow study instructions), had no exacerbation of MS in the past 30 days (with or without disease-modifying drugs), were able to perform the choice stepping reaction time test (Tijsma et al., 2017), were able to walk 10 m without a bilateral mobility aid, had no existing conditions that prevented exercise, and had not been advised by a medical doctor to avoid exercise. Ability to perform the choice stepping reaction time test ensured that participants were capable of undertaking the intervention. All participants recruited to the main iFIMS trial were invited to participate in this sub-study until recruitment was complete. Prior to randomisation in the main iFIMS trial, participants were invited to participate in this sub-study. Sixty-six people agreed to participate and were enrolled, these people completed the iFIMS intervention but also attended an additional pre- and post-intervention assessment as part of this sub-study. Baseline data from 30 participants were previously published to compare proprioception and motor function in people with multiple sclerosis with age- and sex-matched healthy controls (Djajadikarta et al., 2020).

### Participant characteristics

Prior to randomisation, information was obtained on participants’ demographics and type of multiple sclerosis. Average total weekly physical activity (planned and incidental) over the 3-months prior to baseline was measured using the Incidental and Planned Exercise Questionnaire (IPEQ; App Store; Merom et al., 2014). The authors have permission from the copyright holders to use this instrument.

### Experimental protocol

Full details of the iFIMS main study’s intervention, outcome measures, setup, testing procedures, and data processing for the three balance and gait measures are reported in detail elsewhere (Hoang et al., 2024). We report them briefly here.

### Interventions

The *smart*±*step* intervention involved playing home-based step training exergames. A research assistant installed the *smart*±*step* system in each participant’s home, which consisted of a custom-made step mat and a small computer connected to a television or a monitor screen. Participants stood and stepped on the mat to train balance, stepping ability, and cognitive function. Exercise intensity and type were selected by participants as they progressed through six game difficulty levels from ‘Easy’ to ‘Expert’. Participants were instructed to start on an easy level and when ready, progress to more challenging levels. They were encouraged to try to beat their highest score. Goal setting and barriers to training were discussed with participants to encourage compliance and exercise progression. There were eight exergame options available. To ensure that participants played games targeting speed and visuospatial processing, they were required to play two core games each day: ‘Stepmania’, where players stepped on (or avoided) floating arrows with precise timing, and ‘Brick Stacker’, a tile puzzle game where players stepped on arrows to move and rotate geometric shapes as they moved down the screen, to stack them into complete rows (Sturnieks et al., 2019). After this, the other games were made available (Hoang et al., 2024).

The intervention duration was 6 months and participants were asked to exercise for at least 120 min per week, broken into shorter sessions of 20–30 min. Compliance to intervention was monitored *via* daily transfer of data from the *smart*±*step* system to a computer server. If participants did less than 80 min of exercise per week for two weeks, they were contacted by telephone to encourage compliance to exercise.

The control group received usual care. Participants assigned to this group were instructed to continue their usual activities. Participants in both groups were given an educational booklet on exercise and fall prevention for people with MS as education alone does not typically improve fall rates (Gillespie et al., 2012).

### Outcome measures

This sub-study explored effects of the intervention on secondary outcomes of ankle proprioception (movement detection threshold and reaction time) and plantarflexor muscle performance (maximal isometric voluntary torque, voluntary activation using twitch interpolation, twitch torque during electrical stimulation, decrements in voluntary torque, twitch torque and voluntary activation after a sustained isometric contraction, and recovery from these decrements. All testing procedures were performed before and after 6 months of step training or usual care. For both sessions, the same leg was tested, and custom-built chairs were set to the same measurements.

Three outcome measures of balance and gait that were obtained as part of the main trial were included: postural sway during quiet double stance (displacement of the body while standing on a foam mat for 30 s, with the eyes open; Lord, Menz & Tiedemann, 2003), comfortable walking speed (during the 10 Metre Walk Test; 10MWT; Peters, Fritz & Krotish, 2013) and walking endurance (distance walked in the Six Minute Walk Test; 6MWT; ATS Committee on Proficiency Standards for Clinical Pulmonary Function Laboratories, 2002).

### Setup

Participants were instructed to wear the same flat, enclosed, low-top shoes for both sessions. All complied except for nine in the intervention group and five in the control group. Of these, seven intervention participants and two control participants wore similar shoe styles between sessions. Participants who wore different shoes were still included in the analysis. The tested limb was the limb identified by the participant as the one more affected by MS (intervention: 15 right leg; control: 15 right leg). A hand-held goniometer (Baseline 360 degrees) was used to measure voluntary ankle range of motion (Table 1) from maximal plantarflexion to maximal dorsiflexion (neutral position = 0°). The goniometer axis was positioned at the medial malleolus, the stationary arm spanned from the medial malleolus to the medial femoral condyle, and the moving arm was parallel to the base of the footwear.

**Figure 1:**
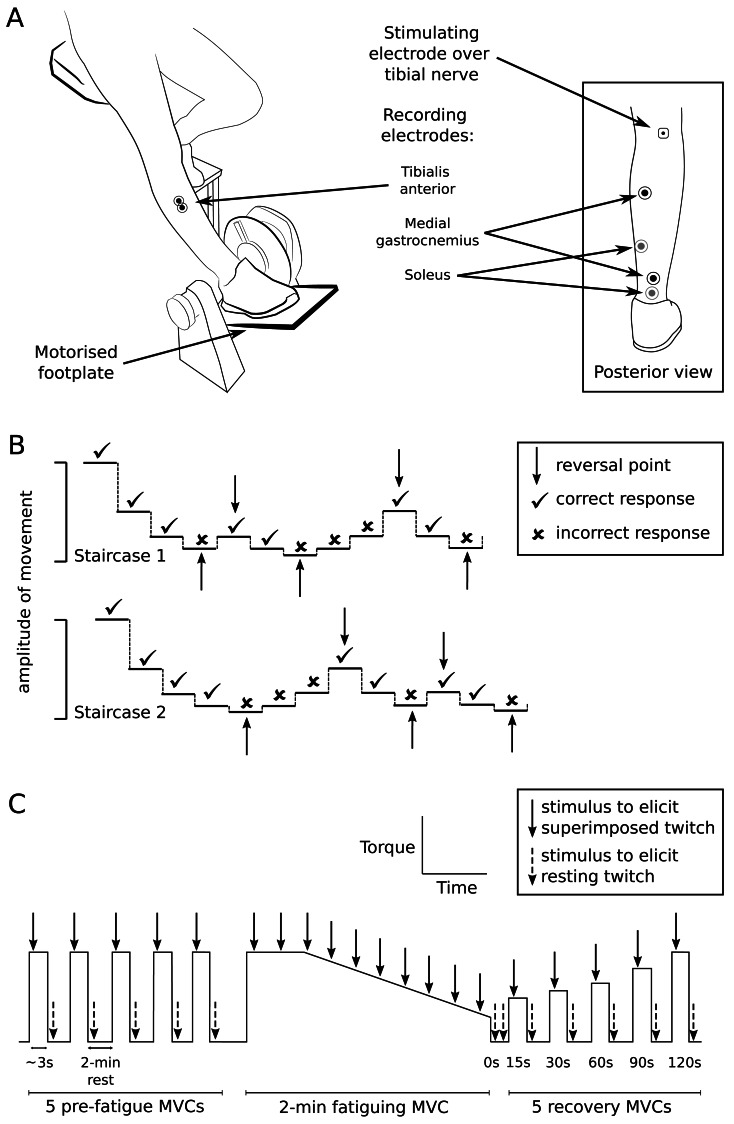
Setup and testing procedures. (A) The custom-built chair and motorised footplate used to test ankle proprioception, and the positions of recording and stimulating electrodes used for proprioception and muscle performance testing. (B) An example of the double staircase method used to assess detection threshold of passive movement about the ankle. Detection threshold was calculated as the average of the movement amplitudes measured at reversal points from both staircases. (C) Outline of experimental protocol for muscle performance testing. The trace represents plantarflexor torque over time (not to scale). To test muscle performance, participants performed 5 pre-fatigue isometric maximal voluntary contractions (MVCs) of the plantarflexors (3 s duration; 2-min intervals), before performing a 2-min sustained MVC (indicated by the plateau and falling slope) to fatigue the plantarflexors. Participants then performed MVCs at 15, 30, 60, 90 and 120 s after the fatiguing contraction to investigate recovery after fatigue. The tibial nerve was stimulated at peak torque to generate superimposed twitches (solid arrows) and immediately after MVCs to generate resting twitches (dashed arrows).

Paired, recording electrodes (20-mm diameter, Ag/AgCl, Conmed, Anaheim, CA, USA) were placed over the tibialis anterior muscle (30-mm, centre-to-centre interelectrode distance) and over the muscle bellies and calcaneal tendon of the plantarflexors (soleus and medial gastrocnemius, using a belly-tendon configuration) (Fig. 1A). Electromyograms from each muscle were amplified (x100–300) and filtered at 16–1,000 Hz (CED 1902 amplifier; Cambridge Electronic Design (CED), Cambridge, UK). During proprioceptive testing, footplate position was recorded using a potentiometer (linearity of  ± 0.25%; Vishay Intertechnology, Malvern, PA, USA) and plantarflexor torque was recorded using a load cell underneath the footplate (Xtran S1W, Applied Measurement Australia, Baywater, Australia). During motor performance testing in a different custom-built device, plantarflexor torque was again recorded using a load cell (Xtran S1W). Electromyograms, torque, and footplate position were sampled at 2,000 Hz (CED Micro 1401-3 converter) and recorded on computer for analysis using Spike2 software (CED). Position changes of 0.01 degree could be identified from the recorded signal.

**Table 1:** Demographic and clinical characteristics of participants taken in the baseline session data are shown as mean (SD) unless otherwise stated.

	Intervention (*n* = 32)	Control (*n* = 33)	*p* value for *t*-test on independent groups
Age (years)	53.44 (13.20)	49.55 (9.91)	*p* = 0.183
Sex (M:F)	8:24	11:22	
Height (cm)	163.62 (7.75)	166.80 (9.70)	*p* = 0.149
Weight (kg)	74.43 (18.63)	74.34 (16.69)	*p* = 0.984
EDSS, median (IQR)	3.5 (3.0 to 4.5)	4.0 (3.5 to 5.5)	*p* = 0.043^*^
MS type			
Relapsing-remitting (n)	22	18	
Secondary-progressive (n)	4	7	
Primary-progressive (n)	3	5	
Unknown (n)	3	3	
IPEQ (h/week)	27.93 (17.2)	21.59 (15.09)	*p* = 0.119
Voluntary ankle ROM (°)			
Plantarflexion	42.89 (12.66)	40.36 (11.81)	*p* = 0.408
Dorsiflexion	15.08 (11.43)	12.42 (11.26)	*p* = 0.349

**Notes.**

EDSS Expanded Disability Status Scale IPEQ Incidental and Planned Exercise Questionnaire MS multiple sclerosis ROM range of motion

* non-parametric Mann Whitney U test.

### Proprioception testing procedures

Participants sat with the foot resting passively on a motorised footplate and the knee straight (Fig. 1A). The footplate position was initially set at midway between voluntary plantarflexion and dorsiflexion. The footplate moved the foot in dorsiflexion-plantarflexion at 13°/s and was controlled by a purpose-built LabVIEW program (LabVIEW v8.2, National Instruments, Austin, TX, USA). A table was positioned over the knees to conceal the legs and feet.

Movement detection threshold was measured using a double-staircase method (Cornsweet, 1962) to assess the smallest passive movement about the ankle that could be detected. Two staircase series were run concurrently, with steps of footplate rotation amplitude in each series alternating in a 1:1 ratio (see Fig. 1B for example). Before each step, the footplate was repeatedly moved up and down several times in a set pattern (a ‘waggle’), so that the movement history and muscle spindle sensitivity before each step was consistent (Proske, Tsay & Allen, 2014). For each step, the footplate randomly moved the foot in dorsiflexion or plantarflexion. Participants were instructed to verbally identify the direction of movement as ‘up’, ‘down’ or ‘I don’t know’. Responses were judged as correct when the reported movement direction matched the direction imposed by the footplate (dorsiflexion = ‘up’, plantarflexion = ‘down’). Participants received no feedback on whether or not responses were correct. For each staircase, a correct response resulted in a 50% decrease in the amplitude of movement of the following step in that staircase, and an incorrect/unidentified response resulted in a 50% increase. Each staircase ended after five reversal points (when the participant’s response changed from correct to incorrect or vice versa).

Reaction time was measured as the shortest time a participant took to respond to a movement at the ankle. The footplate passively moved the foot in dorsiflexion or plantarflexion at random, and participants actively plantarflexed into the footplate as soon as movement was detected. The amplitude of footplate rotation was twice the participant’s detection threshold, to ensure participants had no uncertainty that movement had occurred. The sound of the footplate motor was masked by noise played to participants through headphones. To alert participants that the footplate was about to rotate, a loud “beep” sounded, and the footplate rotated randomly after 520–1,500 ms. Five practice trials were provided, then 25 trials were performed with 5-s intervals between trials.

### Muscle performance testing procedures

On a different testing device, participants were seated with the knee straight, and the foot strapped onto a footplate set at 45° from horizontal. Stimulating electrodes (10-mm diameter, Ag/AgCl, Ambu A/S, Ballerup, Denmark) were placed over the tibial nerve in the popliteal fossa (Fig. 1A) and over the medial femoral condyle. Optimal electrode position over the tibial nerve was first determined using low intensity stimulation (10–20 mA; 1-ms duration; DS7A, Digitimer, Garden City, UK) delivered through a custom-built hand probe. The probe was repositioned over the approximate location of the tibial nerve until the largest plantarflexor response was found. A self-adhesive electrode was attached over the final position of the probe. Stimulation intensity was then progressively increased until maximal plantarflexion twitch torque and maximal peak-to-peak amplitude of the muscle compound action potentials from soleus and medial gastrocnemius muscles were identified. The stimulation intensity used for testing was 120% of that used to obtain maximal responses.

Participants first practised isometric maximal voluntary contractions (MVCs) of the plantarflexors on the footplate until torque was consistent (within 10%) over 3–4 MVCs. Participants were given verbal encouragement and visual feedback to attain maximal effort. Five MVCs (∼3-s duration) were performed, with 2-min rests between contractions to prevent exercise-related decrements in performance (Fig. 1C). During each MVC, the tibial nerve was stimulated at peak plantarflexion torque to generate a superimposed twitch, and immediately after the MVC to generate a resting twitch.

Participants were then instructed to perform a maximal isometric plantarflexion contraction against the footplate for 2 min to fatigue the plantarflexors. Similar fatiguing tasks have been carried out with other muscles in people with MS and in other patient groups (*e.g.*, Neyroud et al., 2017; Schwid et al., 1999; Steens et al., 2012; Wolkorte, Heersema & Zijdewind, 2016). Ten superimposed twitches were elicited during the MVC (∼13 s apart), and two resting twitches (2–3 s apart) were elicited immediately after the fatiguing contraction.

Finally, five MVCs (∼3-s duration) were performed to investigate recovery of motor outcomes following the 2-min sustained contraction. Participants performed MVCs at 15, 30, 60, 90 and 120 s after the fatiguing contraction. Superimposed and resting twitches were elicited during each of the recovery MVCs.

### Protocol deviation

One of our planned proprioception secondary outcomes (ankle joint position sense using a motorised foot plate) was not included in analysis, as the measurement of joint position sense using this device is not a validated technique (Vetter & Mascha, 2017).

### Randomisation

Following the baseline assessment, participants were randomly allocated to the intervention or control group using a central web-based randomisation program. Permuted block randomisation (block sizes: 2, 4, 6, 10) using computer-generated random numbers were applied to form two groups of similar size in the main study. All participants were invited to do the sub-study, and recruitment finished when 66 participants agreed to participate.

### Blinding

To ensure allocation concealment, the software and schedule for randomisation were developed by individuals external to the study team. All baseline assessments, follow-up assessments, and statistical analyses were conducted by investigators blind to participant group allocation.

### Data analysis

Detection threshold values were extracted from Spike2 files using a custom-written CED script. First, a median filter (200-ms window) was applied to smooth the footplate angle signal. Next, the five reversal points in each staircase were identified. The footplate angular movement at each reversal was calculated as the difference between the start position (midway point between voluntary plantarflexion and dorsiflexion) and the final position of the plate. An average of the movement amplitudes at the five reversals was calculated for each staircase, and then a single detection threshold was calculated as the average of both.

Reaction time was measured as the mean time (s) between footplate movement onset and voluntary force production (measured as time at 10% of maximal rising slope of torque increase). Trials that were performed incorrectly were excluded (*e.g.*, if the participant applied force before the footplate was moved). Trials that were >3 standard deviations (SD) from the participant’s mean were also excluded.

Muscle performance outcomes of MVC torque, resting twitch torque and voluntary activation were obtained before (non-fatigued), at the end of a 2-min fatiguing MVC, and during recovery after the fatiguing MVC (Fig. 1C). Non-fatigued muscle performance outcomes were obtained from the largest value across the five non-fatigued MVCs. MVC torque was measured as the mean torque over 200 ms before the superimposed twitch. The amplitude of superimposed and resting twitch torques were measured from twitch onset to peak. Voluntary activation (%) (Herbert & Gandevia, 1999; McKenzie et al., 1992) was calculated as:

\begin{eqnarray*}(1-\text{superimposed twitch/resting twitch})~\times ~100. \end{eqnarray*}

Voluntary activation values that were calculated as negative were set at 0% (*n* = 2 fatigued MVCs included in the analysis). Negative values occur if the resting twitch is smaller than the superimposed twitch because of non-linearity in the measured relationship between the superimposed twitch and voluntary torque at low torques. Such values reflect very poor voluntary activation. Here, use of 0% rather than the non-physiological negative values altered the fall in voluntary activation over the fatiguing contraction from 85.3 to 76.9% (baseline session, intervention participant with concurrent 85% fall in maximal torque) and from 48.6 to 27.7% (baseline session, control participant with concurrent 35% fall in maximal torque). Voluntary activation calculated with superimposed twitches with negative amplitudes (fall in torque after stimulation) were set at 100% (*n* = 4 over all MVCs).

Towards the end of the fatiguing contraction, the mean torque over 200 ms before the final superimposed twitch was measured and expressed as a percentage of non-fatigued MVC torque. The mean torque of the two resting twitches immediately after the fatiguing contraction was expressed as a percentage of non-fatigued twitch torque. The mean resting twitch and final superimposed twitch were used to calculate voluntary activation. The changes in MVC torque, resting twitch torque and voluntary activation were expressed as the differences from prior to the fatiguing contraction to the end of the fatiguing contraction.

Time-to-recovery outcomes of MVC torque, twitch torque and voluntary activation were measured from the five recovery MVCs. MVC torque and twitch torque were expressed as percentages of non-fatigued values, and voluntary activation was expressed as the difference from non-fatigued values. For each outcome of each participant, a third order polynomial was fitted over time and used to determine the time at which the outcome recovered relative to the non-fatigued value. MVC torque and twitch torque recovered if they returned to at least 95% of non-fatigued values. Voluntary activation recovered if it returned to within 5% of the non-fatigued value. Third order polynomials were chosen to model time-to-recovery data as they allowed for non-linear relationships. Plots of individual participant raw and fitted data were inspected. Time-to-recovery data were analysed using custom-written scripts in Python v3.8.

### Statistical analyses

In total, there were two proprioception outcomes (detection threshold, reaction time), nine muscle performance outcomes (MVC torque, twitch torque and voluntary activation and the same parameters after a fatiguing contraction, and time-to-recovery of those outcomes), and three balance and gait outcomes (postural sway, walking speed, and gait endurance).

Distributions of the data were assessed by visual inspection of histograms and boxplots. Proprioception outcomes were not normally distributed, and potential outliers were present (∼8 for detection threshold, ∼6 for reaction time).

We used statistical tests different to those in the original statistical analysis plan (Djajadikarta, 2020), as they were more appropriate.

Students t tests (or Mann Whitney U test) for independent groups were used to compare baseline demographic data. Robust linear regression was used to determine between-group effects of intervention for all proprioception, muscle performance, and balance and gait outcomes using the -rreg- command in Stata (Verardi & Croux, 2009; Vickers & Altman, 2001). Robust linear regression is weighted such that extreme observations influence the overall effect less, compared to non-extreme observations. Data were analysed by intention-to-treat.

A complier average causal effect (CACE) analysis was conducted to estimate the average effect of treatment in participants who complied with the intervention. Compliance to the intervention was measured as the total amount of step time (h) performed for each participant in the intervention group, expressed as a percentage of total intervention time. Participants in the control group did not have access to the intervention, and so performed 0 h of intervention. The CACE estimates the mean effect of treatment in participants who undertake 100% of the intervention if allocated to the intervention group and 0% of the intervention if allocated to the control group (Angrist & Imbens, 1995; Fairhall et al., 2017). The CACE was estimated with instrumental variable regression using the -ivregress- command with the two-stage least squares estimator in Stata. The instrument was the randomly allocated intervention. Compliance to the intervention was entered as a continuous variable.

Time-to-recovery outcomes were analysed using mixed effects parametric survival models with the -mestreg- command in Stata (Cleves et al., 2008). Survival models account for non-normal and right-censored data (*i.e.,* when participants had not recovered after the 120-s period of observation).

Statistical analysis was conducted with IBM SPSS Statistics software (v25, IBM, Armonk, NY, USA) and Stata (v16; StataCorp, College Station, TX, USA). All de-identified data and computer code used for analysis are available from the public repository (http://www.osf.io/gazfw).

## Results

**Figure 2:**
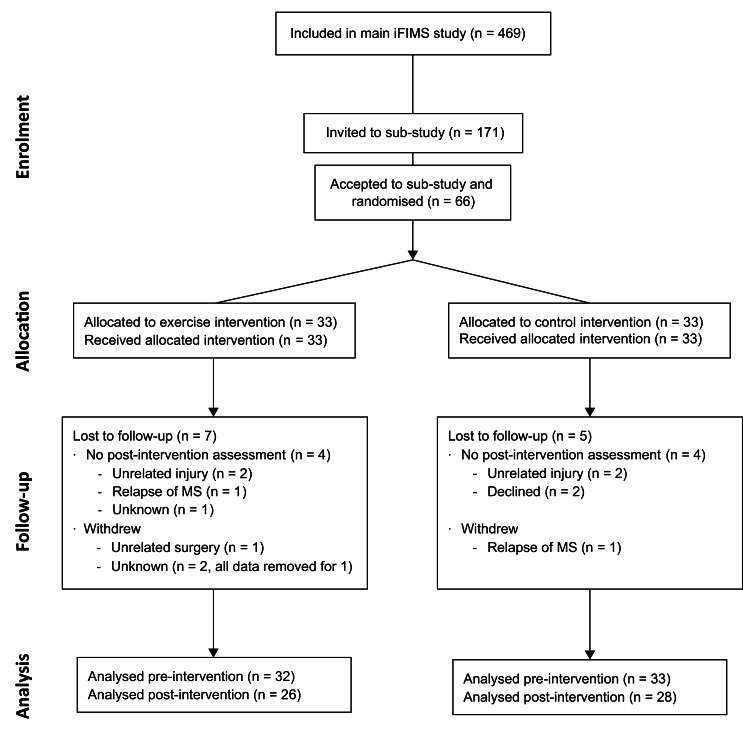
CONSORT flow diagram.

Baseline demographic and clinical characteristics are shown in Table 1. All participant’s were recruited between 5 August 2016 and 15 October 2018. The trial ended when the sample size was reached. Follow-up for all participants was completed by 15 May 2019, and occurred between 168 and 255 days (median: 207 days) after baseline. Progression of participants through the study is shown in the CONSORT (Schulz et al., 2010) flow diagram (Fig. 2). The total number of incidental and planned hours of physical activity (IPEQ; mean (SD)) recorded for the 3 months prior to baseline are shown in Table 1, and the number of hours recorded for the final 3 months of the intervention were 29.5 (12.8) h/week for intervention and 21.7 (14.8) h/week for control.

Participants in the intervention group exercised with the *smart*±*step* system for an average of 60.3 (53.5) min/week during the 6-month trial, with six intervention participants reaching the target time of 2 h/week. The mean (SD) total exergame playing time in this group was 23.5 (22.1) h. No adverse events related to step training were reported.

**Figure 3:**
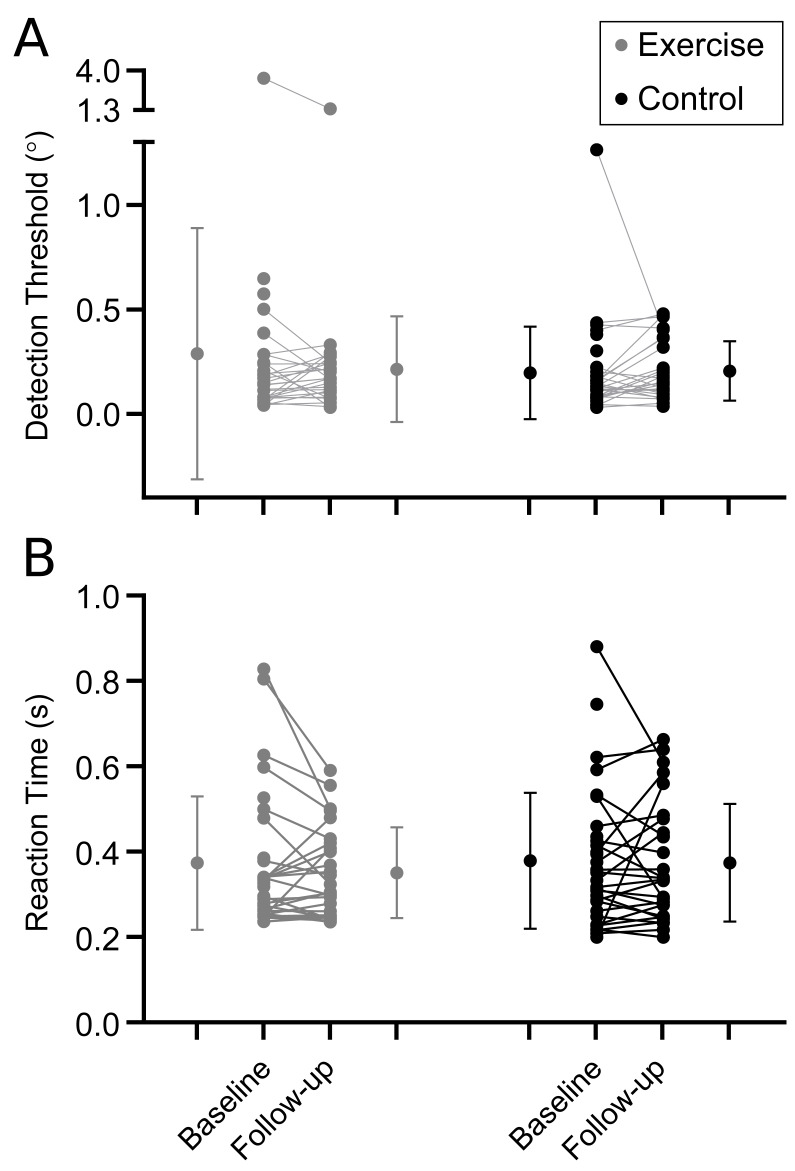
Proprioceptive outcomes. Individual baseline and follow-up data for (A) detection threshold and (B) reaction time to movement about the ankle. Within group means (SD) for baseline and follow-up measures are indicated to the left and right of the individual participant data.

**Figure 4:**
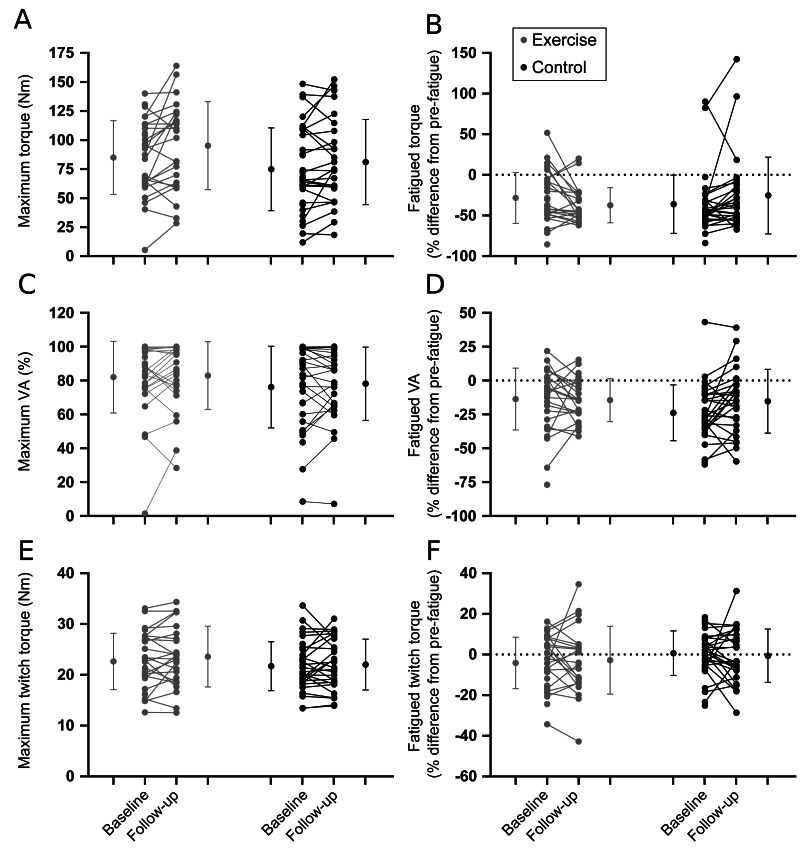
Muscle performance outcomes. Individual baseline and follow-up data for pre-fatigue (left panel) and fatigued (right panel) outcomes. (A, B) Maximal voluntary contraction torque. (C, D) Voluntary activation. (E, F) Resting twitch torque. Horizontal dotted lines represent no change from pre-fatigue values. Within group means (SD) for baseline and follow-up measures are indicated to the left and right of the individual participant data.

Results are shown in Table 2. In general, there were no differences between intervention and control groups for proprioception (Fig. 3; Table 2), muscle performance (Fig. 4; Table 2), or postural sway and gait endurance (Supplemental 2; Table 2). There was a borderline statistically significant increase in walking speed in the intervention group, and a borderline statistically significant decrease in MVC torque after the fatiguing sustained contraction in compliers with the intervention compared to control (Supplemental 2; Table 2). However, these effects are small in magnitude and unlikely to be clinically relevant. There was no difference in time-to-recovery between intervention and control groups for MVC torque (between group hazard ratio 0.47, 95% CI [0.13–1.69], *p* = 0.25), resting twitch torque (0.46, 95% CI [0.17–1.29], *p* = 0.14) and voluntary activation (0.80, 95% CI [0.37–1.72], *p* = 0.57; Supplemental 3).

**Table 2:** Within-group summary statistics and between-group comparisons of proprioception, muscle performance, and balance and gait outcomes. Within-group means of fatigued outcomes are % differences from pre-fatigue values, and negative values indicate that the fatigued value is smaller than the pre-fatigue value. Positive values of between-group mean differences indicate that the exercise group mean is greater than the control group mean.

	Intervention		Control		Between-group mean difference ITT (95% CI)	Between-group mean difference CACE (95% CI)
	Baseline mean (SD)	Follow-up mean (SD)	Baseline mean (SD)	Follow-up mean (SD)		
Proprioception						
Detection threshold (°)	0.29 (0.60) *n* = 32	0.21 (0.25) *n* = 26	0.20 (0.22) *n* = 33	0.21 (0.14) *n* = 28	−0.01 (−0.07 to 0.04) *p* = 0.63	−0.05 (−0.16 to 0.05) *p* = 0.34
Reaction time (s)	0.37 (0.16) *n* = 32	0.35 (0.11) *n* = 26	0.38 (0.16) *n* = 32	0.37 (0.14) *n* = 28	0.00 (−0.03 to 0.04) *p* = 0.87	−0.04 (−0.14 to 0.04) *p* = 0.30
Non-fatigued						
Torque (Nm)	84.9 (31.8) *n* = 29	95.2 (38.0) *n* = 24	74.8 (35.7) *n* = 32	81.1 (36.7) *n* = 27	5.9 (−3.4 to 15.2) *p* = 0.21	19.1 (−4.9 to 43.1) *p* = 0.12
Resting twitch (Nm)	22.6 (5.6) *n* = 29	23.6 (6.0) *n* = 24	21.7 (4.8) *n* = 32	22.0 (5.0) *n* = 27	0.5 (−1.3 to 2.3) *p* = 0.58	1.0 (−2.5 to 4.4) *p* = 0.58
VA (%)	82.0 (21.2) *n* = 29	82.9 (20.0) *n* = 24	76.1 (24.1) *n* = 32	78.1 (21.7) *n* = 27	4.8 (−0.7 to 10.3) *p* = 0.08	7.1 (−5.0 to 19.1) *p* = 0.25
End of fatiguing contraction						
Torque (%)	−28.3 (31.4) *n* = 29	−37.3 (21.6) *n* = 23	−36.1 (35.9) *n* = 32	−25.4 (47.2) *n* = 27	−9.3 (−19.2 to 0.7) *p* = 0.07	−44.3 (−86.5 to −2.1) *p* = 0.04^*^
Resting twitch (%)	−4.1 (12.7) *n* = 29	−2.8 (16.6) *n* = 23	0.6 (11.0) *n* = 32	−0.6 (13.1) *n* = 27	−0.7 (−7.4 to 5.9) *p* = 0.82	−1.4 (−16.4 to 13.6) *p* = 0.85
VA (%)	−13.7 (22.8) *n* = 29	−14.4 (15.9) *n* = 23	−23.8 (20.6) *n* = 32	−15.3 (23.6) *n* = 27	−6.0 (−15.5 to 3.5) *p* = 0.21	−12.1 (−34.1 to 9.9) *p* = 0.28
Balance and gait						
Postural sway (mm)	324 (180) *n* = 29	220 (118) *n* = 26	280 (212) *n* = 31	215 (221) *n* = 26	42 (−34 to 117) *p* = 0.28	−42 (−235 to 151) *p* = 0.67
10MWT (m/s)	1.58 (0.77) *n* = 32	1.66 (0.43) *n* = 26	1.41 (0.51) *n* = 33	1.44 (0.58) *n* = 28	0.16 (0.02 to 0.30) *p* = 0.03^*^	0.25 (−0.18 to 0.68) *p* = 0.26
6MWT (m)	425 (140) *n* = 32	432 (127) *n* = 26	387 (139) *n* = 33	361 (156) *n* = 28	36 (−2 to 75) *p* = 0.07	74 (−2 to 150) *p* = 0.06

**Notes.**

CACE Complier average causal effect EDSS Expanded Disability Status Scale IPEQ Incidental and Planned Exercise Questionnaire ITT Intention-to-treat effect N number of participants VA voluntary activation 6MWT 6 Minute Walk Test 10MWT 10 Metre Walk Test

* indicates statistical significance.

## Discussion

Contrary to our hypothesis, this exploratory study in a subgroup of participants from a larger randomised controlled trial showed that 6 months of step training using the *smart*±*step* computerised exergame playing system did not improve ankle proprioception or plantarflexor muscle performance outcomes in people with multiple sclerosis. A small increase in walking speed was observed in the intervention group compared to control (Hoang et al., 2024); however, complier average causal effect analysis revealed no average effect of treatment on this outcome. Step training did not improve proprioception, muscle performance, or any balance and gait outcome.

The lack of improvement in ankle proprioception and plantarflexor muscle performance in this sub-study are not surprising given the outcomes of the main study. The primary outcome of the full randomised controlled trial (*n* = 469) was the rate of falls over the first 6 months of the study (Hoang et al., 2024). This primary outcome and other falls-related outcomes were negative. Falls were unchanged by the intervention. About half (*n* = 210) of participants underwent tests of balance, gait and muscle strength before and after the six-month intervention. No changes were seen in postural sway, walking speed or strength of the knee extensor or hip flexor muscles. The only positive findings were improved performance of aspects of choice stepping reaction time tasks (small to medium effect sizes, d = −0.24 to −0.44; Hoang et al., 2024).

Other factors may also have influenced the results of the sub-study. Baseline ankle proprioception and plantarflexor muscle performance of the first 30 participants were compared to those of age- and sex-matched controls (Djajadikarta et al., 2020). This revealed a lack of substantial impairment of proprioceptive detection threshold in people with MS (Djajadikarta et al., 2020) and suggests there may be little room for improvement in the measure. With clinical testing of proprioception (identification of passive movement of a digit or of the direction of movement), impairment is typically reported in about one-third of the limbs tested (*e.g.*, see Table 2 of Fukutake et al., 1998; Scherder et al., 2018). More recently, decrements in contralateral position matching or ipsilateral repositioning at the ankles have been reported (Iandolo et al., 2020; Ozkan et al., 2023). Movement detection is the most basic proprioceptive test as it relies only on a signal from brief activation of proprioceptive afferents reaching the cortex. Matching the position of a passive ankle and foot by actively moving the other foot (Iandolo et al., 2020) or actively repositioning a foot to a previous passive position (Ozkan et al., 2023) requires more processing of signals such that the different afferent firing from passive and active muscles can be understood as the same joint position. In addition, matching the left and right foot position requires transfer of signals between cerebral hemispheres while repositioning requires the initial position to be memorised. Thus, different proprioceptive tests may be affected differently by pathology.

Proprioceptive reaction time, in which participants detected an imposed movement about the ankle and responded to that movement with a voluntary plantarflexion, is impaired in people with MS (Djajadikarta et al., 2020). Even so, step training did not change this outcome. By contrast, in the main study, movement time for the choice stepping reaction time task, where participants step onto one of four squares when the target lights up, was improved by an average of ∼40 ms after step training (Hoang et al., 2024). The differences between these two tasks include the more complex choice *versus* the simple reaction task, a visual *versus* a proprioceptive stimulus and standing *versus* sitting posture. The former requires shifts of posture and centre of gravity before a step can be made whereas the latter does not. Consequently, the more complicated choice stepping task had a response time of ∼1.28 s compared to the ∼0.38 s for the proprioceptive reaction time (Hoang et al., 2024). Nonetheless, simple reaction times, which involved pressing a key in response to visual stimuli, have been improved in people with MS by 12 sessions of visuo-motor practice (Dana, Rafiee & Gholami, 2019). This suggests that either practice in the current study was not sufficiently intense to result in improvements or was not sufficiently specific, in that the reaction task used a proprioceptive ‘go’ signal while the step training used visual cues to direct stepping. For both proprioceptive outcomes, the mean difference between the intervention and control groups is close to zero with relatively narrow confidence intervals that encompass the null effect. Therefore, we can be confident in inferring that there is no effect of step training on these outcomes, compared to usual care.

Like the proprioceptive outcomes, plantarflexor muscle performance was unchanged by step training. Impairment of motor performance through impaired neural drive to the muscles has been reported by studies examining the first dorsal interosseous, knee extensor and ankle dorsiflexor muscles in people with multiple sclerosis (for review see Mamoei et al., 2020). Similarly, we have previously reported that plantarflexor twitch sizes in people with multiple sclerosis are similar to healthy controls, but neural drive (voluntary activation) to the plantarflexor muscles is substantially reduced (∼79% in the current study *versus* ∼90% in healthy controls) with a consequent reduction in maximal voluntary torque (Djajadikarta et al., 2020). People with MS also display greater central fatigue (an exercise-induced reduction in voluntary activation; Gandevia, 2001) than healthy controls during fatiguing plantarflexor contractions (Djajadikarta et al., 2020). However, none of these measures were altered by step training. The lack of change in resting twitch size, which is independent of central nervous system impairment as it is evoked by peripheral nerve stimulation, suggests that the intervention did not produce muscle adaptations. In addition, there were no changes in maximal voluntary torque or voluntary activation. That is, step training did not improve the ability of the nervous system to drive the plantarflexor muscles maximally. Finally, the intervention did not improve muscle strength or motor performance during fatiguing exercise, and did not change time-to-recovery. Thus, neither strength nor endurance was improved by six months of home-based step training.

Previous studies show that exercise training can improve walking ability, balance, proprioception, and muscle performance in people with MS (Gunn et al., 2015; Pearson, Dieberg & Smart, 2015; Snook & Motl, 2009; Sokhangu et al., 2021). Reina-Gutiérrez et al. (2023) reviewed 72 trials of various types of training in people with MS and used network meta-analysis to find that aerobic training (15 studies) was effective at improving cardiorespiratory fitness (effect size 0.6), and resistance or combined training (total of 29 studies) improved muscular fitness (effect sizes 0.66 and 0.77 respectively) with improvement plateauing after 30–50 sessions. However, only one of the reviewed studies examined plantarflexor muscle strength (Fimland et al., 2010; *n* = 7 per group) and reported increased plantarflexor maximal torque after 15 sessions of maximal strength training over 3 weeks. As the increased torque was accompanied by increased maximal EMG and V waves, an increase in neural drive to the muscle is likely (Fimland et al., 2010). Despite the small sample size, the study suggests that it is possible for sufficiently intense training to improve maximal strength of the plantarflexors in people with MS. It is unclear why improvements were not seen in the current trial. It is possible that the sample size was too small, and the study was underpowered to detect effects. However, the estimates of between-group differences were precise. Otherwise, the total duration, volume (*i.e.,* min/week), and intensity of training may have been insufficient to produce adequate muscle and/or proprioceptive adaptations. Moreover, training with the *smart*±*step* system is classed as ‘exercise at home’ in the planned exercise category of the IPEQ, and the total number of hours of incidental and planned physical activity recorded for the final 3 months of the intervention were similar to those recorded at baseline for both intervention and control groups (Hoang et al., 2024). Therefore, it is possible that participants in the intervention group replaced their usual exercise with step training, rather than performing the intervention in addition to their usual exercise, as instructed. Substitution of step training for similar intensity exercise would make effects on strength and endurance unlikely.

Studies delivering interventions over 3–10 weeks suggest that the balance and gait benefits of the *smart*±*step* system may be enhanced by increasing the number of stepping directions used during training (Ganesan, Skias & Aruin, 2021), combining training with education and behavioural therapies that encourage goal setting and self-efficacy (Hayes et al., 2017), or performing exergames on an unstable surface, to enhance the postural demands of training (Kramer, Dettmers & Gruber, 2014). Direct benefits for muscle strength are likely to require more intense or long-lasting muscle activity. For example, maximal plantarflexor strength increase by ∼33% in young sedentary men after concentric-eccentric heel raises performed twice a week for 8 weeks with up to 70 heel raises in one session (Nakamura et al., 2025), and by ∼38% in people with stroke after 100 heel raises five times a week for 6 weeks (Jung et al., 2020).

A previous study investigating a pilot version of the *smart*±*step* system with a similar sample size (*n* = 50) to this trial showed improvements in several measures of balance, stepping ability, and gait in people with multiple sclerosis (Hoang et al., 2016). It is possible that differences in participant compliance and study methodology may have contributed to contrasting results. For example, compliance in the intervention group in this study averaged 60 min per week, whereas compliance in the previous trial was 71 min per week (Hoang et al., 2016). Further, the inclusion of six additional games in the iFIMS trial could have reduced training efficacy, and the shorter duration of the intervention (12 weeks) used for the previous trial could help explain differences in results (Hoang et al., 2016). While greater effects from shorter trials would generally be unexpected, recent meta-analyses on the effects of exercise training on walking mobility, balance, and falls in people with multiple sclerosis found that a shorter duration but higher volume (*i.e.,* more min/week) of training may be more effective (Gunn et al., 2015; Snook & Motl, 2009). Any potential improvements in outcomes measured here may be counteracted by the natural disease progression over extended periods, or technique and motivation may decline towards the end of a longer intervention. Nevertheless, the same training was effective at reducing the risk of falls in older people without multiple sclerosis (incidence rate ratio = 0.74 in comparison to controls) (Sturnieks et al., 2024). These older adults had better adherence than the participants with MS and completed an average of ∼80 min training per week over 12 months. However, despite the improved falls rates and longer training durations, secondary balance and gait outcomes did not improve (Sturnieks et al., 2024). Thus, home-based step training can reduce falls in some populations but this may occur without specific improvements in proprioceptive or motor performance.

### Limitations

As a sub-study of a trial aimed at reducing falls through home-based step training, the current study was not specifically designed to improve ankle proprioception or muscle performance. Therefore, the lack of change in these parameters does not necessarily indicate that they cannot be improved by other exercise regimens. Adherence to the goal of 120 min of training per week was low and the main study was ineffective at reducing falls (Hoang et al., 2024). In addition, the number of participants in the sub-study was low. However, the lack of change in ankle proprioceptive and muscle performance is consistent with the secondary outcomes of the main trial which showed no improvement in physical functions like balance, gait, and knee and hip strength (Hoang et al., 2024). Finally, the baseline performance of proprioceptive detection threshold did not differ from that of healthy control participants (Djajadikarta et al., 2020) so a test that revealed more impairment may have offered more chance for improvement with training.

## Conclusion

In people with multiple sclerosis, the *smart*±*step* computerised exergame playing system used in the iFIMS clinical trial did not improve the secondary outcomes of ankle proprioception and muscle performance when compared to usual care.

##  Supplemental Information

10.7717/peerj.20354/supp-1 Supplemental Information 1 CONSORT checklist

10.7717/peerj.20354/supp-2 Supplemental Information 2 Outcomes of balance and gait at baseline and follow-up sessions Individual baseline and follow-up data for **(A)**the total sway path of bipedal stance on a foam mat for 30 s, (**B)** walking speed during the 10 Meter Walk Test (10MWT) and **(C)**the furthest distance achieved in the 6 Minute Walk Test (6MWT). Within group means (SD) for baseline and follow-up measures are indicated to the left and right of the individual participant data.

10.7717/peerj.20354/supp-3 Supplemental Information 3 Recovery time of muscle performance outcomes after a 2-min sustained maximal contraction Individual participant data for baseline and follow-up recovery time(s). Note that data are not available for participants who had not recovered by 120 s after the fatiguing contraction**.**Graphs show data for **(A)**maximal voluntary contraction (MVC) torque (exercise: 15/29 participants at baseline and 15/22 at follow-up had not recovered by 120s; control: 18/32 at baseline and 13/25 at follow-up had not recovered by 120s), (**B)**voluntary activation (exercise: 9/29 at baseline and 7/23 at follow-up had not recovered by 120s; control: 10/32 at baseline and 4/25 at follow-up had not recovered by 120s), and **(C)**resting twitch torque (exercise: 4/29 at baseline and 3/23 at follow-up had not recovered by 120s; control: 1/32 at baseline, and 1/27 at follow-up had not recovered by 120s).

10.7717/peerj.20354/supp-4 Supplemental Information 4 Demographic and pre and post intervention measures for control and intervention groups These measures include balance and gait assessments as well as specialised assessments of ankle proprioception and plantarflexor muscle performance.

## Additional Information and Declarations

### Data Availability

The following information was supplied regarding data availability: The data are available in the Supplemental File and at OSF: Djajadikarta, Zoe, and Joanna Diong. 2025. “Effects of Home-based Step-training on Ankle Proprioception and Muscular Performance in People with Multiple Sclerosis: A Sub-study Analysis.” OSF. July 9. www.osf.io/gazfw.

### Clinical Trial Registration

The following information was supplied regarding Clinical Trial registration: ACTRN12616001053415.
