# Supplementary figures and images for: Proprioception and muscle performance unchanged by in-home step training in multiple sclerosis: secondary outcomes analysis
Source: PeerJ. 2025 Dec 19;13:e20354. doi: 10.7717/peerj.20354 (PMC12721106; doi:10.7717/peerj.20354)

**A**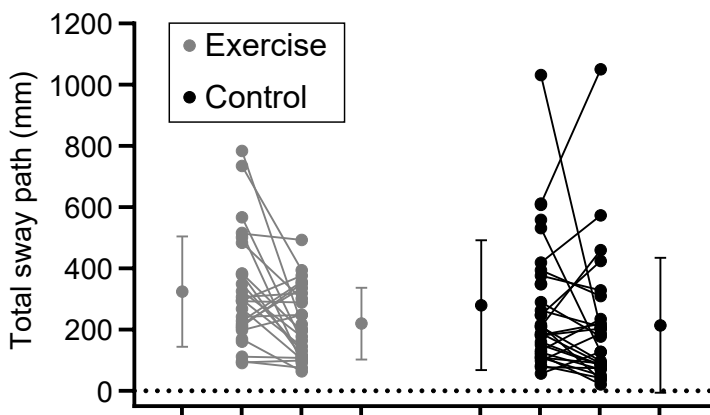**B**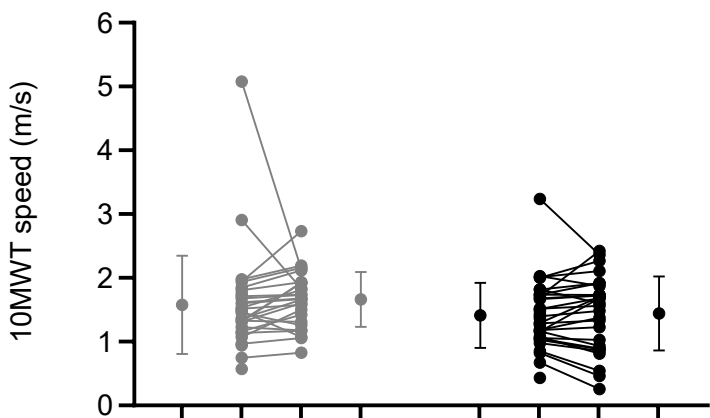**C**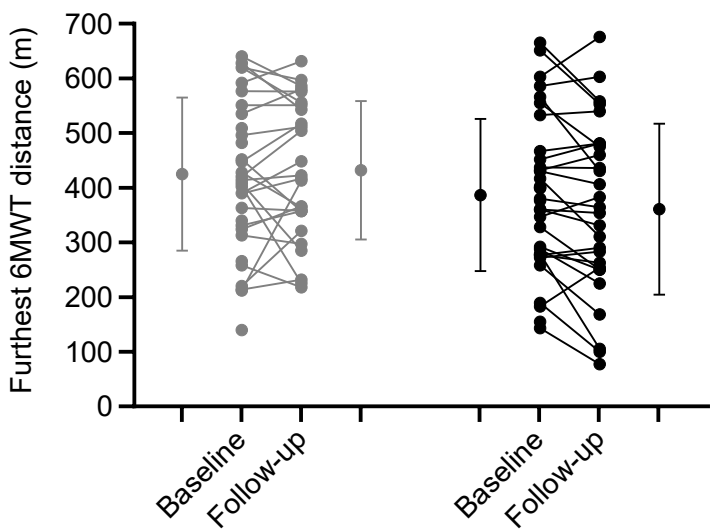

Supplement: Supplemental Information 2 — Individual baseline and follow-up data for (A)the total sway path of bipedal stance on a foam mat for 30 s, (B) walking speed during the 10 Meter Walk Test (10MWT) and (C)the furthest distance achieved in the 6 Minute Walk Test (6MWT). Within group means (SD) for baseline and follow-up measures are indicated to the left and right of the individual participant data. [file peerj-13-20354-s002.pdf]

**A**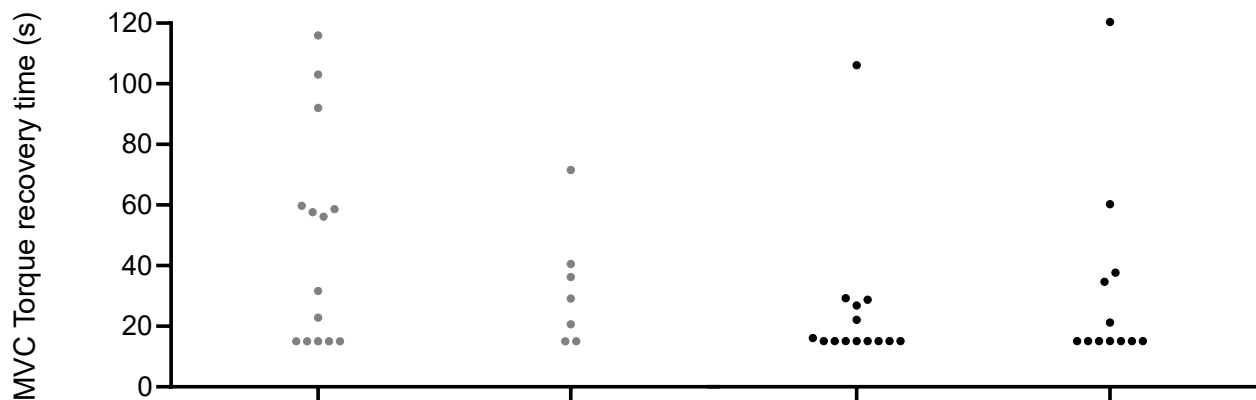**B**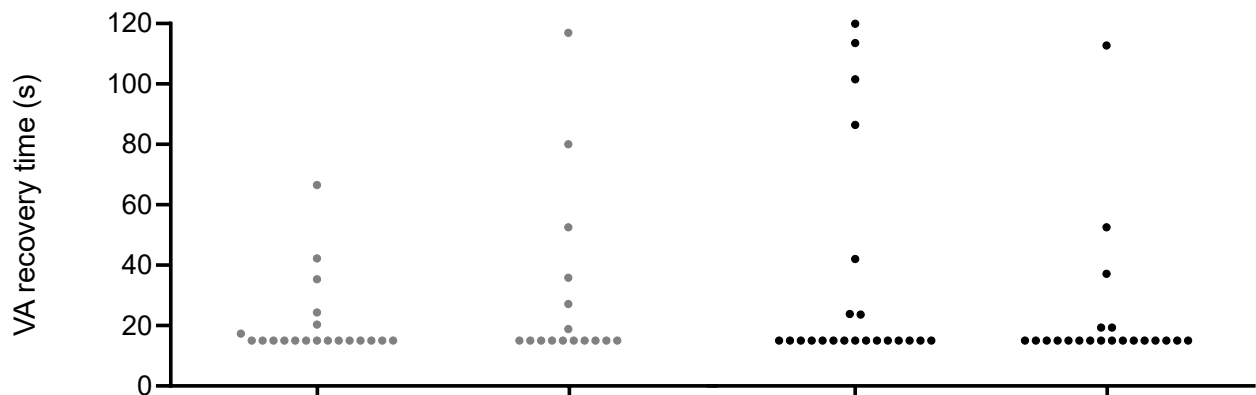**C**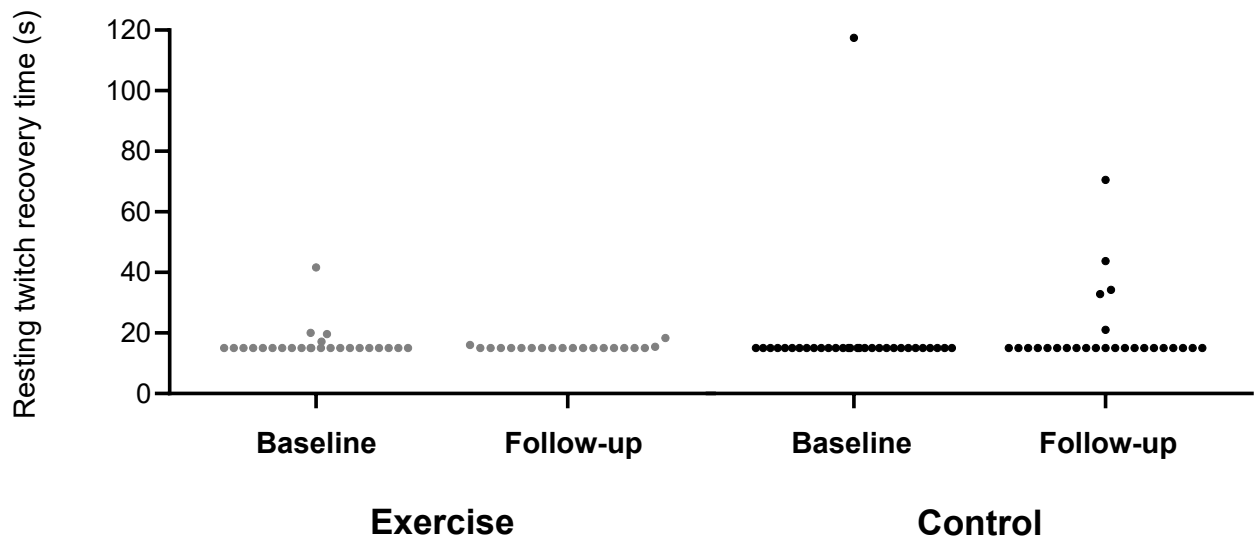

Supplement: Supplemental Information 3 — Individual participant data for baseline and follow-up recovery time(s). Note that data are not available for participants who had not recovered by 120 s after the fatiguing contraction.Graphs show data for (A)maximal voluntary contraction (MVC) torque (exercise: 15/29 participants at baseline and 15/22 at follow-up had not recovered by 120s; control: 18/32 at baseline and 13/25 at follow-up had not recovered by 120s), (B)voluntary activation (exercise: 9/29 at baseline and 7/23 at follow-up had not recovered by 120s; control: 10/32 at baseline and 4/25 at follow-up had not recovered by 120s), and (C)resting twitch torque (exercise: 4/29 at baseline and 3/23 at follow-up had not recovered by 120s; control: 1/32 at baseline, and 1/27 at follow-up had not recovered by 120s). [file peerj-13-20354-s003.pdf]
